# Impact of workplace gymnastics on quality of life of general services assistants in a hospital institution

**DOI:** 10.47626/1679-4435-2022-1034

**Published:** 2024-09-24

**Authors:** Fabio Tormem, Natalia Cristina de Oliveira, Sônia Maria Marques Gomes Bertolini, Regiane da Silva Macuch

**Affiliations:** 1 Departamento de Saúde, Faculdade Santa Maria da Glória, Maringá, PR, Brazil; 2 Mestrado em Promoção da Saúde, Centro Universitário Adventista de São Paulo, São Paulo, SP, Brazil; 3 Programa de Pós-Graduação em Promoção da Saúde, Universidade Cesumar, Maringá, PR, Brazil

**Keywords:** exercise, housekeeping hospital, health promotion, exercício físico, serviço hospitalar de limpeza, promoção da saúde

## Abstract

**Introduction:**

The adoption of worker care by organizations has been subject of several studies. Hospitals are strategic spaces for health promotion actions to be developed.

**Objectives:**

To analyze the effects of a workplace gymnastics program on the quality of life of hospital workers.

**Methods:**

This was an exploratory longitudinal analytical study. For data collection, the following instruments were employed: sociodemographic questionnaire, assessment of labor gymnastics, World Health Organization Quality of Life instrument-Abbreviated version and Nordic Musculoskeletal Questionnaire, both applied before and after the workplace gymnastics program. The program for general service workers was carried out for 15 weeks, 3 times a week.

**Results:**

After the intervention, a significant improvement was observed in the physical health (p = 0.0001), psychological (p = 0.0001), social relationships (p = 0.0220), environment (p = 0.0001) and general quality of life domains (p = 0.039). However, for environment and general quality of life domains, the increase in the respective average scores was not enough to change the domain classes from fair to good. As for the effect of the intervention on painful symptoms, verified by the Nordic Musculoskeletal Questionnaire, a decrease in pain was observed in the neck (p = 0.0455) and knee (p = 0.0455) regions.

**Conclusions:**

Workplace gymnastics provided positive results for workers and, therefore, its practice should be encouraged, as it improves in quality of life and health promotion at work.

## Abstract (pt)

**Introdução:**

A adoção de cuidados com o trabalhador por parte das organizações tem sido tema de diversos estudos. Os hospitais constituem espaços estratégicos para que ações de promoção de saúde sejam desenvolvidas.

**Objetivos:**

Analisar os efeitos de um programa de ginástica laboral na qualidade de vida de trabalhadoras de uma instituição hospitalar.

**Métodos:**

Trata-se de um estudo analítico exploratório longitudinal. Para a coleta de dados, foram utilizados um questionário sociodemográfico, uma avaliação da ginástica laboral, o World Health Organization Quality of Life instrument-Abbreviated version e o Nordic Musculoskeletal Questionnaire. Eles foram aplicados antes e após a realização do programa de ginástica laboral. O programa para as trabalhadoras de serviços gerais foi realizado por 15 semanas, três vezes por semana.

**Resultados:**

Após a intervenção, observou-se uma melhora significativa para os domínios físico (p = 0,0001), psicológico (p = 0,0001), de relações sociais, (p = 0,0220), de meio ambiente (p = 0,0001) e de qualidade de vida geral (p = 0,039). Todavia, para os domínios de meio ambiente e qualidade de vida geral, o incremento nos respectivos escores médios não foi suficiente para modificar as classes dos domínios de regular para boa. Quanto ao efeito da intervenção na sintomatologia dolorosa, verificada com a utilização do Nordic Musculoskeletal Questionnaire, foi observada diminuição da dor nas regiões do pescoço (p = 0,0455) e joelho (p = 0,0455).

**Conclusões:**

A ginástica laboral proporcionou resultados positivos para as trabalhadoras, e, portanto, sua prática deve ser estimulada, uma vez que auxilia na melhoria da qualidade de vida e na promoção da saúde no trabalho.

## INTRODUCTION

Most of our day is spent in the workplace. Therefore, in order to achieve a better performance in our professional activity, this workplace should be as pleasant and healthy as possible. However, hospitals, where patients’ health is restored, are often a stressful and unsatisfactory environment, generally leading workers to feel physically, mentally, and emotionally drained.^1^

Concern for the well-being of workers in their workplace is relevant and necessary in every organization. Companies should associate their concern for workers’ quality of life (QoL) with the degree of satisfaction they get from performing their duties at work. Health is a universal right, guaranteed in Article 189 of the Constitution of the Federative Republic of Brazil. Article 7 establishes that “workers’ rights [...] include [...] the mitigation of risks inherent to work,”^2^ and that this should be done through occupational medicine.

Programs that seek to improve QoL can and should include labor gymnastics (LG), given the countless benefits provided. No matter the form or method of exercise, breaks during the working day bring benefits in terms of preventing musculoskeletal symptoms and those related to QoL, such as reducing stress, mental strain, and fatigue.^3^

LG is defined as a set of bodily exercises in the workplace. It usually lasts between 10 to 15 minutes and aims to compensate for the physical and psychological wear and tear on workers, restore their productive energies and prevent occupational illnesses as a way of promoting occupational QoL.^4^

This program, known internationally as Workplace Physical Activity Intervention, consists of specific physical exercises to compensate for the muscle groups that are most demanded during work duties. Thus, LG can be defined as a physical and motor activity to compensate for wear and tear and aid disorder prevention.^5^

This study aimed to analyze health conditions and the impact of an initiative and practice of health promotion through exercise in general service workers at a hospital in Maringá, Paraná, Brazil, to promote QoL and improve musculoskeletal symptoms. Similar studies have been conducted with nurses, nursing technicians, and nutrition and dietetics professionals at a hospital, including the studies by Hipólito et al.^6^ and Souza et al.^7^ However, few studies have been conducted with general service workers. In this context, there is 1 study with cleaning and urbanization workers,^8^ but not at hospitals.

## METHODS

This is an applied, analytical, longitudinal exploratory study with a quantitative approach. The study’s target population was women working in the general service sector in a hospital. All women in the general service sector were personally invited to take part in the study. The sample consisted of general service workers from four different sectors of the hospital: cleaning, laundry, sanitizing, and packaging. The sample included workers who had been working in these sectors for at least 6 months and had been in the job market for at least 1 year.

The study was conducted in a high-complexity hospital in a municipality in the northwest of Paraná, Brazil. The intervention was conducted in an open-air setting; however, in the face of rainy days, we decided to use an indoor environment of the hospital, which was airy and spacious, since the study was conducted during the COVID-19 pandemic.

We used 4 survey instruments to collect data: a sociodemographic questionnaire and some information on professional training; the World Health Organization Quality of Life instrument-Abbreviated version (WHOQOL-Bref); the Nordic Musculoskeletal Questionnaire (NMQ); and an assessment of the LG program. In addition to these tools, conversation circles were held once a week to obtain information for a qualitative analysis. However, this proved unsuccessful because most participants (95%) dropped out at this stage.

The sociodemographic questionnaire was made up of 11 questions, involving data on age, sex, marital status, weight, and height (from which the body mass index [BMI] was calculated), education, job title/function, length of service in the job title/role, working hours, and whether they had another job.

The WHOQOL-Bref survey consists of 26 questions, 2 of which deal with overall QoL, the responses follow a Likert scale model (from 1 to 5, the higher the score, the better the QoL). The remaining 24 questions (facets) are divided into 4 domains: physical health, psychological, social relationships, and environment. The result is presented as an average (1 to 5) per domain.^9^

The version of the NMQ used was validated for the Portuguese population by Mesquita et al.^10^. The participants evaluated the LG program. The questionnaire consisted of five closed questions and three open questions and sought to collect data on the perception of the workers regarding the program.

Data were collected according to the availability of the participants, on predetermined days and at predetermined times. The questionnaires were completed individually, although the investigator accompanied them to clarify any questions.

The LG program for the general service workers was held for 15 weeks, 3 times a week. The program consisted of a warm-up, stretching exercises, small group dynamics, and resistance, strength, and relaxation exercises.

The data obtained was analyzed using Statistical Analysis Software (SAS), version 9.4, from a database compiled using Excel. The data was described using absolute and percentage frequency tables. The differences between the mean scores for the WHOQOL-Bref domains were tested using the Wilcoxon test for paired data.

McNemar’s test was used to compare the differences in frequencies for the categories of domains established by the WHOQOL-Bref and for the categories established by the NMQ (pain or no pain), for each body part before and after the LG program (last 7 days). All tests used a 95% confidence level.

This study was submitted to the board of directors of the hospital participating in the study and to the Research Ethics Committee at Cesumar University (UNICESUMAR) for analysis and approval (opinion No. 5.008.805, Certificate of Submission for Ethical Appraisal 518873217.0000.5539). In order to guarantee the confidentiality and anonymity of the data collected, participants were asked not to self-identify on the questionnaires and, after completing them, to place them in an envelope the investigator had provided.

## RESULTS

Initially, 32 workers agreed to participate in the study; however, 12 did not respond to the questionnaires before starting the LG program, nor did they attend the sessions. Therefore, the study 20 workers who worked during the day shift.

The age of the participants ranged from 20 to 50 years (mean 38.75±7.94). The age group between 31 and 40 years comprised the majority (45%), followed by 35% between 41 and 50 years. As for marital status, 45% of the participants were single, 40% married, and 15% divorced. The majority (70%) had a high school diploma (Table 1).

**Table 1:** Sociodemographic data of the participants, hospital located in the northwest of Paraná, Brazil, 2022

Profile	n	%
Age group (years)		
20 to 30	4	20.00
31 to 40	9	45.00
41 to 50	7	35.00
Marital status		
Single	9	45.00
Married/partnered	8	40.00
Divorced	3	15.00
Education		
Elementary school	6	30.00
High school	14	70.00
Job/role characteristics		
Occupation		
Cleaning assistant	13	65.00
Packer	1	5.00
Sanitation worker	2	10.00
Laundry assistant	4	20.00
Length of service (years) in the job		
Less than 1	6	30.00
1 to 3	5	25.00
4 to 6	4	20.00
7 to 10	3	15.00
More than 10	2	10.00
Holds another job		
No	19	95.00
Yes	1	5.00
Daily workload (hours)		
8	18	90.00
12	2	10.00
Weekly workload (hours)		
36	1	5.00
44	18	90.00
60	1	5.00
Body mass index		
Normal	7	35.00
Overweight	6	30.00
Obese	7	35.00

Most participants (65%) worked as cleaners and had done so for less than 1 year (30%); had no other job (95%); and worked 44 hours a week, 8 hours a day (90%). Before the intervention, 30% of the participants were overweight and 35% were obese.

The participants perceived their QoL before the LG program as fair in all domains. The highest mean score (3.87;standard deviation [SD] = 0.9138) was for the social relationships domain, and the lowest mean score (3.11; SD = 0.7348) was for the environment domain.

After the LG program, a significant improvement was observed for the QoL domains, namely: physical health (p = 0.0001), psychological (p = 0.0001), social relationships (p = 0.0220), environment (p = 0.0001), and overall QoL. The increase in the respective mean scores was not sufficient to change the respective domain classes (Table 2).

**Table 2:** Mean score per domain and QoL, SD, classification, and p-value for the paired Wilcoxon test observed for the participants of a hospital located in the northwest of the state of Paraná, Brazil, 2022

Domains	Before LG		After LG		p-value
	Mean (SD)	Rating	Mean (SD)	Rating	
Physical health	3.44 (0.7657)	Fair	4.38 (0.6083)	Good	0.0001*
Psychological	3.70 (0.6343)	Fair	4.24 (0.5861)	Good	0.0001*
Social relationships	3.87 (0.9138)	Fair	4.20 (0.7601)	Good	0.0220*
Environment	3.11 (0.7348)	Fair	3.88 (0.6502)	Fair	0.0001*
QoL	3.58 (0.9072)	Fair	3.95 (0.6469)	Fair	0.0239*

SD = standard deviation; QoL = quality of life.

*Significant at 95% confidence level.

Before the intervention, 85% of the participants felt that the environment domain (WHOQOL-Bref) was poor or fair (Table 3).

**Table 3:** McNemar’s test distribution of frequencies and p-value according to domains, observed for participants at a hospital located in the northwest of Paraná, Brazil, 2022

Domains	LG program				
	Before LG		After LG		p-value
	n	%	n	%	
Overall QoL					
Poor/fair	10	50.00	4	20.00	0.0143*
Good/very good	10	50.00	16	80.00	
Physical health					
Poor/fair	15	75.00	4	20.00	0.0009*
Good/very good	5	25.00	16	80.00	
Psychological					
Poor/fair	13	65.00	5	25.00	0.0047*
Good/very good	7	35.00	15	75.00	
Social relationships					
Poor/fair	9	45.00	5	25.00	0.0455*
Good/very good	11	55.00	15	75.00	
Environment					
Poor/fair	17	85.00	9	45.00	0.0047*
Good/very good	3	15.00	11	55.00	

LG = labor gymnastics; QoL = quality of life.

*Significant at the 95% confidence level.

After the intervention, the perception that the environment domain was poor or fair was only 45% (p = 0.0047). The participants changed their perception of the physical health domain significantly (p = 0.0009). Before the intervention, 75% of the participants felt that their physical QoL was poor or fair. After the intervention, their perception dropped to 20%. For all other domains, table 3 shows a significant improvement: overall QoL (p = 0.0143), psychological (p = 0.0047), and social relationships (p = 0.0045).

After the intervention, participants observed no improvement in pain relief in the elbow (90%) and hip/thigh (75%). Only for the neck (p = 0.0455) and knee (p = 0.0455) did pain relief show significant differences. Before the LG program, 35% of the participants felt pain around the neck, dropping to 15% after the LG program. Regarding knee pain, pain relief dropped from 60% to 40% of participants. The rate of participants who reported feeling pain, discomfort, or disturbance in the upper back increased from 15% to 30%. For the other parts, although we observed a downward trend in the proportion of participants who reported feeling pain, discomfort, or disturbance, differences were not significant (Table 4).

**Table 4:** McNemar’s test distribution of frequencies, reported pain or no pain, and p-value per body part, before and after the LG program (last 7 days) according to participants at a hospital located in the northwest of Paraná, Brazil, 2022

Body part	Pain, discomfort, or disturbance								p-value
	Before LG				After LG				
	No		Yes		No		Yes		
	n	%	n %		n	%	n	%	
Neck	13	65.00	7	35.00	17	85.00	3	15.00	0.0455*
Shoulders	13	65.00	7	35.00	16	80.00	4	20.00	0.0833
Upper back	17	85.00	3	15.00	14	70.00	6	30.00	0.0833
Elbow	18	90.00	2	10.00	18	90.00	2	10.00	
Lower back	10	50.00	10	50.00	14	70.00	6	30.00	0.1573
Wrists/hands	13	65.00	7	35.00	14	70.00	6	30.00	0.3173
Hip/thigh	15	75.00	5	25.00	15	75.00	5	25.00	
Knee	8	40.00	12	60.00	12	60.00	8	40.00	0.0455*
Ankle/foot	13	65.00	7	35.00	15	75.00	5	25.00	0.1573

LG = labor gymnastics.

*Significant at 95% confidence level

Although most participants (95%) felt that the LG program was necessary, only 1 said that she participated in the LG program frequently, while the others participated occasionally. Only 15% of the participants practiced physical activities other than the LG program (Table 5).

**Table 5:** Evaluation of the LG program among participants at a hospital located in the northwest of Paraná, Brazil, 2022

Items	n	%
Participated in the LG program		
Sometimes	4	20.00
Often	16	80.00
Considered LG		
Unnecessary	1	5.00
Necessary	19	95.00
Participated in other physical activity		
No	7	35.00
Sometimes	10	50.00
Often	3	15.00
Degree of benefit from the LG program		
None/few	2	10.00
Some	2	10.00
Good/Many	15	75.00
Meaning of the LG program for the participant		
Relaxation/leisure/pleasure	16	80.00
Stimulation/prevention	3	15.00
Prevention and relaxation	1	5.00
After LG, what is your mood for the working day?		
In a good mood	8	40.00
Good/very good	8	40.00
Work happily	1	5.00
Did not answer	3	15.00
Changes perceived in the body after joining the LG program		
Did not answer	6	30.00
Pain relief/relaxation/very good	7	35.00
Lively/energized/less tired	5	25.00
Much pain	1	5.00
No changes	1	5.00
Suggestions to improve the LG program		
None/did not answer	16	80.00
Increase frequency/availability in the early evening	3	15.00
Have a permanent location	1	5.00

LG = labor gymnastics.

Most participants (75%) had a positive perception of the benefits of LG. The majority (80%) perceived it positively (relaxation/leisure/pleasure). After LG, 85% of the participants felt good (lively/energized, good/very good, or worked happily), and 15% of the participants had no opinion. After admission to the LG program, 60% of the participants noticed positive bodily changes (pain relief/relaxed/very good/lively/energized/less tired). The participants found it difficult (80%) to give an opinion or make suggestions when requested (Table 5).

Therefore, participants’ perceived their QoL had improved after the LG program in all domains. Improvement in pain relief was only observed for the neck and knee. Participants reported feeling pain, discomfort, or disturbance in the upper back. For the other parts, no significant difference was observed. The participants acknowledged the importance of the LG program and had a positive perception of the degree to which the LG program had benefited their physical well-being.

## DISCUSSION

In light of the relevance of the evaluation results, the sociodemographic profile of the sample analyzed was first surveyed. The concept of QoL is understood to be subjective, multidimensional, and influenced by various factors related to education, economics, and social and cultural aspects, as well as being directly linked to the individual’s perception of their own condition.^11^

Most participants in this study were between the ages of 20 and 30 (20%) and 31 to 45 (45%) years, totaling 65%. They are therefore considered to be young adults, as explained by Gonçalves,^12^ who believes that at this age individuals are at the peak of their physical abilities, as they have more muscle tissue, more calcium in their bones, more brain mass, better visual acuity, greater aerobic capacity, and a more efficient immune system.

Caliari et al.,^13^ in a study involving nursing professionals, found that the average age of women was 36.4±8.8 years. Therefore, the result found in this study (average 38.75±7.94) is very close to that of Caliari et al.^13^

As for their educational background, most participants (70%) had completed high school or not, while 30% had completed elementary school, in contrast to the results found by Silva et al.^14^ in their study with waste collectors, in which only 9.3% had completed high school. On the other hand, 45% were single and 40% were married. In their study, the percentage of married people was 69.8%.

In this respect, Gonçalves^12^ states that during this life span, experimentation is initially allowed before the responsibilities of a stable adult are assumed, noting that traditional tasks such as finding a stable job and developing long-term affective relationships have been postponed until after the age of 30 and emphasizing that the job chosen is affected by their academic background, intelligence, family values and resources, and their personality.

A second variable analyzed was BMI, which showed that only 30% were within the normal range, while 30% were overweight and 35% were obese. Obesity is a serious public health problem and is prevalent worldwide. This demonstrates the importance of weight loss programs aimed at improving occupational health, productivity, self-esteem and, consequently, their QoL.^8^

This study also revealed that 65% of the participants were cleaning assistants, of whom the highest percentage had been working in this position for less than 1 year, working a 44-hour week and an 8-hour day. Most had no other job. According to the Classificação Brasileira de Ocupações (CBO, Brazilian Classification of Occupations), the profile and duties of a cleaning assistant involve good physical capacity and willingness to do heavy or tiring tasks if necessary agility in their work, good organizational skills, among others.^15^

The application of the WHOQOL-Bref before the LG program showed that workers’ self-assessment was classified as fair (mean score between 3 and 3.9), with the highest mean score in the social relationships domain and the lowest mean score in the environment domain. In a study with intensive care unit nurses, 35% rated their QoL as fair, 22.5% as poor and 5% as very poor, and the domain with the lowest score was also the environment.^16^ This domain is related to safety, condition of the physical environment, opportunity to acquire new information and skills, money for essentials, leisure, housing, transportation, and access to health services.

The lower score for the environment domain is not surprising, since these workers come into contact with physical, biological, ergonomic, mechanical, and chemical agents on a daily basis when performing their duties. In addition, the positions involved in this sector have weaknesses such as low income and lack of specific knowledge, which have an impact on QoL.^17^

WHOQOL-Bref after the LG program found a significant improvement in self-perception of QoL in all domains. In this respect, Souza et al.^7^ state that LG provides physiological, psychological, and social benefits to workers. The physiological benefits include increased blood circulation in the muscular structure due to increasing heart rate, leading to improved oxygenation of the muscles and tendons, and reducing lactic acid accumulation. It also improves applied muscle and joint mobility and flexibility, reducing inflammation and trauma, posture and motor coordination, and reduces unnecessary tension and effort when performing tasks. Since workers learn to recruit only the muscles they need, this makes it easier to adapt to the job and reduces muscle fatigue, physical stress, and repetitive strain injury/work-related musculoskeletal disorders (RSI/WMSDs); in other words, the general state of health is improved.

In terms of musculoskeletal symptoms such as pain, discomfort, or disturbance, we observed they have been significantly reduced in the neck and knee, while in the back they have increased and in other parts they have shown a tendency to decrease. Dartora & Santos^17^ argue that musculoskeletal symptoms in general service workers particularly affect the neck, shoulder blades, upper limbs, and knees, as these are the most overloaded parts of the body when performing a task. These pain sites in the shoulders were also identified in a study by Silva & Salete.^18^ Dartora & Santos^17^ emphasize that the factors associated with pain in these sites include repetitive movements, carrying and lifting loads, turning and bending, and high occupational physical demands.

As a result, pain was easier to locate and accurately identified, which, according to Duarte et al.^19^ is because the NMQ has illustrated content, which facilitates proper understanding and identification of the location of the symptoms. In this sense, Lima^20^ explains that pain can change with physical activities, postures, state of attention, emotions, environmental temperature, and humidity, considering that pain is an experience described in terms of sensory, motivational, and cognitive characteristics and often has emotional sequelae.

As for the evaluation of the LG program, most participants perceived its benefits, saying that they began to feel good and to work more willingly and happily. They also noticed positive bodily changes, including pain relief. However, in the open-ended questions, the participants did not give their opinion, which according to the researchers’ inference, may be related to the short time span between the end of the LG program and their return to work.

## CONCLUSIONS

The results have been analyzed and significant differences were found between the initial and final assessments of QoL in all domains, indicating a positive effect of LG, confirmed by the results obtained by the NMQ at the physical, psychological, and social levels. This shows that the LG program can contribute to improving the participants’ QoL both inside and outside the workplace. The participants acknowledged the benefits achieved through the LG program in their evaluations. As for the degree of adherence to the LG program, most workers frequently participated in the sessions.

Therefore, the LG program provided positive results for workers, and its practice should be encouraged. To this end, it is important to publicize, encourage, and involve company leaders to embed LG as part of organizational culture. Therefore, this study has made a major contribution to the interdisciplinary field of health promotion, showing that the practice of targeted physical activities, as is the case of the LG program, is relevant to the QoL and health promotion of hospital workers.

It is also important to stress the need to conduct further investigations with workers in the general services sector of hospitals because of the scarcity of studies with this population, whose work involves a great deal of physical effort, exhausting working hours, unfavorable physical working conditions, among other aspects that can contribute to emotional and physical exhaustion and stress, directly affecting their occupational QoL.
